# Effectiveness of Pro-Safe Work Training (PSWT) and perception of workplace safety among undergraduate nursing students: a mental health perspective

**DOI:** 10.1186/s12912-026-04930-3

**Published:** 2026-06-23

**Authors:** Payel Sarkar, Binil Velayudhan, Vani Lakshmi, Renjulal Yesodharan

**Affiliations:** 1 Manipal College of Nursing, Manipal Academy of Higher Education, Manipal, India; 2 Prasanna School of Public Health, Manipal Academy of Higher Education, Manipal, India

**Keywords:** Workplace violence, Violence prevention, Occupational health, Workplace safety, Nursing students, Safety management, Training program

## Abstract

**Introduction:**

Workplace violence in healthcare is a significant concern, particularly for nurses and nursing students. Nursing students are particularly vulnerable due to limited exposure to clinical settings and poor preparedness. Preventive safety measures are essential to improve both competence and protection from violence. Unlike conventional training programmes that focus on knowledge acquisition, Pro-Safe Work Training (PSWT) integrates behavioural rehearsal, de-escalation strategies, and self-protection skills.

**Objectives:**

The study aimed to evaluate the effectiveness of PSWT in improving awareness of workplace violence prevention and perception of workplace safety among undergraduate nursing students.

**Methods:**

This study used a one-group pretest-posttest design to evaluate intervention effectiveness. Fifty students were selected using purposive sampling. Data collection instruments included a demographic proforma, an awareness questionnaire, and a safety perception scale. The PSWT covered safety measures such as de-escalation techniques, self-defence, and communication skills.

**Results:**

The PSWT significantly improved awareness of workplace violence (*p* = 0.001) while the change in perception of workplace safety was not statistically significant (*p* = 0.24). Post-training, awareness levels increased, with 90% of participants scoring above 21, and the proportion of participants with high perception increased from 14% to 34%, indicating a meaningful positive trend.

**Conclusion:**

PSWT is effective in enhancing awareness; however, improving perception may require sustained and multi-component interventions. The findings are limited by the absence of a control group, small sample size, and single-institution setting.

**Supplementary Information:**

The online version contains supplementary material available at https://doi.org/10.1186/s12912-026-04930-3.

## Introduction

Healthcare workers (HCWs) are often affected by violence in their workplace. When it comes to the psychiatric setting, workplace violence (WPV) can become a serious occupational issue that has significant implications for both the patient and the healthcare provider. It also affects the standard of care and increases the service cost [1, 2]. The Occupational Safety and Health Administration (OSHA) considers WPV as any physical assaults, intimidation, harassment, or other disruptive behaviour that occurs at work [3]. WPVs may cause both physical injuries and psychological consequences, with high rates of stress [4] and other traumatic sequelae for the healthcare provider, which can lead to compassion fatigue, reduced productivity [5], disengagement from patient care, patient falls, medication errors, and overall lower quality of care [6, 7].

A systematic review and meta-analysis conducted by Iozzino et al. (2015) [2] reported that approximately one in five patients admitted to acute psychiatric care units may exhibit violent behaviour. A review reported that 30%-76% of HCWs working in psychiatric settings have been assaulted during their careers [8]. Male patients, a diagnosis of schizophrenia, substance use, and a lifetime history of violence are the major individual factors that lead to violent behaviour. Conversely, the perceived threat of WPVs may lead to increased use of restrictive interventions [9] such as seclusion, restraint, and enforced medication, which patients often describe as traumatic [10].

In hospital environments, workplace safety and injury rates are closely tied to employees’ perceptions of safety practices. Positive employee perceptions, along with employee, supervisor, and administrative participation in safety support activities, are associated with lower work-related injury and illness rates. This, in turn, creates a positive and safe, supportive work climate [11] for the patient and healthcare workers [12]. Psychiatric nurses experience a high level of WPV, resulting in psychological distress [13]. There is also a significant correlation between violence and their quality of life. Srivastava et al. (2025) [14] reported that 97.5% of Indian nurses had experienced verbal abuse in their workplace. Although most hospitals have safety protocols, workplace violence remains a significant issue in the nursing profession [15]. These issues also extend to nursing students. Nursing students, who are often unfamiliar with clinical settings and hospital cultures [16], are more vulnerable to workplace violence, which can undermine their confidence, self-esteem, and career decisions [17]. The nursing facilitators are often unable to prevent workplace violence incidents directed at nursing students [18].

Research indicates that nursing students are vulnerable to workplace violence due to limited clinical experience and hierarchical clinical environments [19]. These WPVs can affect clinical decision-making, career progression, learning opportunities, self-confidence [20] and cause long-standing emotional distress among nursing students [21, 22]. While incidents of violence against healthcare workers and students are common globally, underreporting remains a challenge. In India, several high-profile incidents highlight the growing prevalence of workplace violence, emphasising the need for greater awareness, preventive measures, and improved safety protocols in healthcare settings to protect both students and professionals [23]. Although studies on workplace violence among healthcare workers have been conducted globally, research remains limited, and underreporting is a major challenge [24], particularly in studies involving nursing students in India. According to the findings from a study by Srivastava & Kumar, (2025) [25]; there is a substantially low reporting rate despite a high prevalence of violence among healthcare personnel. The low reporting rate of workplace violence is a result of limited awareness of the reporting procedures [26].

Several factors contribute to workplace violence, including ineffective patient-provider communication, lack of cultural competence training of HCWs, limited experience, overcrowding, resource scarcity, long waiting times, inadequate security measures, dissatisfaction with health services, high cost of care, the negative media influence, and other socio-behavioural factors [27, 28].

Despite the availability of workplace violence prevention programs, most interventions primarily focus on knowledge enhancement, with limited emphasis on modifying safety perceptions. The conceptual basis of this study is grounded in experiential learning theory, which emphasises active engagement, simulation, and skill-based training as effective strategies for improving behavioural competence among healthcare professionals [29]. The safety climate frameworks suggest that perception of workplace safety is shaped by organisational, social, and systemic factors rather than knowledge alone [30]. Evidence suggests that educational interventions improve knowledge outcomes; however, changes in perception and behaviour often require sustained and multi-level strategies [31] and are often influenced by organisational culture, prior exposure, and psychological readiness. This highlights the need for targeted, structured interventions addressing both awareness and perception, improved workplace safety policies, WPV reporting procedures [32] and continuous monitoring to support nurses’ and student nurses’ well-being and job satisfaction.

## Methods

The present study employed a quantitative research approach to assess the effectiveness of Pro-Safe Work Training (PSWT) on nursing students’ awareness of workplace violence prevention and perceptions of workplace safety. A one-group pretest-posttest design was used for the study due to feasibility constraints within the academic setting and to provide preliminary evidence on intervention effectiveness. Although this design does not include a control group, it enables within-subject comparison and reduces inter-individual variability. A purposive sampling technique was used to recruit 50 BSc Nursing students from a nursing institute in the Udupi district. This approach was selected based on accessibility and feasibility. However, the use of purposive sampling and single-institution setting may limit generalizability of findings. Sample size estimation was performed for a paired (pretest–posttest) design assuming a 95% confidence level and 80% power. Based on the mean difference of 3 and a standard deviation of 7 (effect size ≈ 0.43) derived from a preliminary observation within the study setting, the minimum required sample size was calculated to be 43 participants. After accounting for a 10% attrition rate, the adjusted sample size was 48. The final sample size of 50 participants therefore exceeded this requirement and was considered adequate to detect a statistically meaningful difference.

Data collection tools included a demographic proforma, the Awareness Towards Prevention of Workplace Violence Questionnaire (PWPVQ), and the Perception of Workplace Safety Likert Rating Scale (PWPS). The tools are provided in Supplementary file 1*.* The 12-item demographic proforma was designed to collect background information including age, gender, clinical hours completed during the fifth semester, type of hospital where clinical postings were conducted, residential status, area of residence, prior training on workplace safety and violence prevention, preparedness to manage workplace violence, and whether the participant had ever experienced such incidents.

The awareness questionnaire (PWPVQ) was developed by the authors to evaluate participants’ awareness regarding strategies for preventing violence in healthcare settings. It consisted of 30 multiple-choice questions spanning several key domains, including the definition and identification of workplace violence, prevalence and associated risk factors, preventive and intervention strategies, environmental and cultural considerations, as well as procedures for reporting and follow-up. The minimum and maximum possible scores were zero and 30, respectively, and the scores were categorized as poor knowledge (scores 10 and below), average knowledge (scores between 11 and 20), and good knowledge (scores 21 and above).

The Perception of Workplace Safety (PWPS) is a Likert rating scale developed by the authors to assess participants’ perceptions of safety within the clinical workplace environment. The scale included 30 items covering six critical domains: physical safety, security measures, organisational policies and procedures, psychological safety, education and training, and communication and teamwork. Responses were rated to yield a total score ranging from 30 to 150, with interpretation categories as follows: scores of 111 and above indicating a high perception of workplace safety, 71 to 110 indicating a moderate perception, and scores of 70 or below reflecting a low perception of workplace safety.

The reliability of the tools was tested, with Cronbach’s alpha values of 0.912 and 0.92 for the awareness and perception scales, respectively, indicating high internal consistency.

### Pro-safe work training (PSWT)

The Pro-Safe work training package was developed to address key safety competencies, including de-escalation techniques, self-defence, and communication skills. The PSWT module is a structured four-hour instructional program aimed at preparing nursing students to identify, manage, and prevent incidents of violence in healthcare environments. An initial segment was conducted through guided discussions to encourage student interaction, followed by sessions on workplace violence, such as aggression by patients and visitors, interpersonal conflicts among staff, and behavioural risks linked to mental illness or substance abuse. Topics such as active listening, expressing empathy, reading body language, and managing conflicts effectively were also discussed. Verbal and non-verbal de-escalation methods, emphasising techniques like maintaining a calm tone, using appropriate distance, acknowledging the other person’s emotions, and setting clear personal boundaries, were also covered through instructor-led demonstrations. A hands-on session focusing on self-defence techniques and creating a realistic practice environment through roleplays facilitated active participation and experiential learning. Topics such as documentation of violent incidents, legal frameworks and protections available to medical professionals were also part of the PSWT.

## Results

### Demographic characteristics

**Table 1:** Distribution of sample characteristics

Sample characteristics	Frequency (*n*)	Percentage (%)
**Gender**		
Male	8	16.0
Female	42	84.0
**Residential status**		
Day scholar	8	16.0
Hostel Residents	42	84.0
**Area of residence**		
Urban	44	88.0
Rural	6	12.0
**Have you had any previous workplace safety training?**		
Yes	4	8.0
No	46	92.0
**Do you have Previous workplace violence prevention training?**		
No	50	100.0
**Are you prepared to handle workplace violence?**		
Yes	18	36.0
No	32	64.0
**Have you ever been the victim of workplace violence?**		
Yes	1	2.0
No	49	98.0

N = 50 (Total sample); n = number of participants; % = percentage; each category is mutually exclusive and percentages sum to 100% within each variable

The demographic data presented in Table 1 indicate that males have a slightly higher average age of approximately 20.4 years, whereas females have a mean age of 20. The difference between the two is low, indicating that both groups are similar in age. However, males are slightly older than female participants. Most of the participants were female (84%), and only 16% were male. Most of the students (84%) stayed in hostels, while 16% were day scholars. In terms of residence, 88% were from urban areas and 12% were from rural areas. A large percentage of participants (92%) had never received prior workplace safety training, and none (100%) had received workplace violence prevention training. In terms of preparedness, 36% were confident they could manage workplace violence, while 64% were not confident enough to handle workplace violence. Only 2% reported being a victim of workplace violence, with the majority (98%) claiming no such experience.

### Awareness about prevention of workplace violence

Data distribution was assessed prior to analysis. As awareness scores were not normally distributed, median and interquartile range were used for reporting. The participants had a median awareness score of 24 (interquartile range [IQR] = 3.75) (Table 2). The possible score ranged from 0 to 30, while the observed scores ranged from 16 to 28. The findings indicate a relatively high level of baseline awareness among participants, with scores clustered toward the upper range of the scale. The narrow interquartile range suggests limited variability, reflecting a homogeneous level of knowledge across the group. This may be attributed to prior theoretical exposure or curriculum-based learning.

### Perception of workplace safety

Perception scores were normally distributed and are therefore presented as mean and standard deviation. The mean perception score was 100.16 ± 12.81, with a median of 101 (IQR = 13) (Table 2). The observed scores ranged from 78 to 144, within a possible range of 30 to 150. The results suggest a moderate level of perceived workplace safety among participants. The wider variability in scores indicates differences in individual experiences and perceptions, likely influenced by clinical exposure, environmental factors, and personal readiness to manage workplace violence. This highlights the need for targeted interventions to improve consistency in safety perception. Furthermore, the increase in the proportion of participants with high perception (14% to 34%) suggests a meaningful positive shift. Although not statistically significant, this indicates that the intervention may have initiated perceptual changes that could become significant with repeated or longitudinal training.

**Table 2:** Descriptive statistics of pretest scores for awareness about prevention of workplace violence and perception of workplace safety (*N* = 50)

Variables	Mean	SD	Median	IQR	Minimum-Maximum	
					Obtained Range	Possible Range
Awareness about Prevention of Workplace Violence	23.16	2.75	24	3.75	16–28	0–30
Perception of Workplace Safety	100.16	12.81	101	13	78–144	30–150

N = total sample size; SD = standard deviation; IQR = interquartile range; values are presented as mean ± SD and median (IQR); obtained range indicates observed scores and possible range indicates theoretical score limits

### Effectiveness of Pro-Safe Work Training (PSWT)

**Table 3:** Pre–post comparison of awareness and perception scores using Wilcoxon signed-rank test and paired t-test (*N* = 50)

Variables	Pretest	Post-test	Test Statistics	*p*-value
Awareness About Prevention of Workplace Violence	24.0 (3.75)	25.0 (3.00)	W = 154	0.001*
Perception of Workplace Safety	100.16 ± 12.81	103.28 ± 16.06	t = -1.17	0.24

N = total sample size; IQR = interquartile range; SD = standard deviation; W = Wilcoxon signed-rank test statistic; t = paired t-test statistic; values are presented as median (IQR) for awareness and mean ± SD for perception; *p < 0.05

**Fig. 1:**
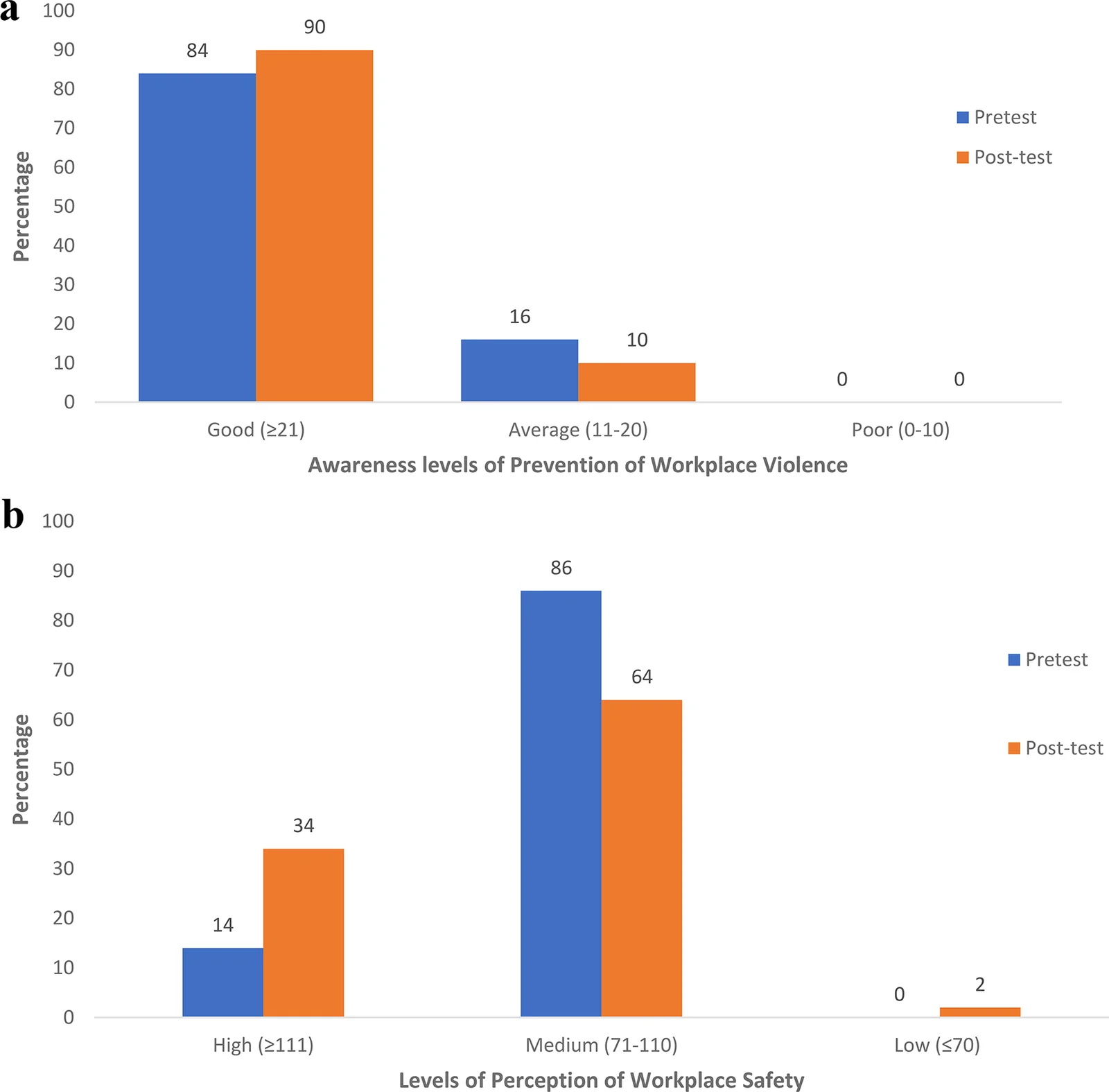
(**a**) Levels of awareness towards the prevention of workplace violence before and after the Pro-Safe Work Training. (**b**) Levels of perception of workplace safety before and after the Pro-Safe Work Training

The effectiveness of Pro-Safe Work Training (PSWT) was assessed based on the changes in the dependent variables, namely awareness about prevention of workplace violence and perception of workplace safety from pretest to post-test. Appropriate parametric and non-parametric tests were applied based on data distribution. A Wilcoxon signed-rank test was used to assess differences in awareness before and after PSWT. The median values of awareness scores increased from 24.0 (*IQR* = 3.75) in the pretest to 25.0 (*IQR* = 3.00) in the post-test (*W* = 154, *p* = 0.001). This indicates a statistically significant improvement in awareness. These findings indicate that PSWT was effective in improving participants’ awareness. The details are presented in Table 3. Additionally, the proportion of participants with good awareness increased from 84% in the pretest to 90% in the posttest. No participants were classified under poor awareness following the intervention. These findings further support an improvement in awareness following PSWT (Fig. 1a).

Similarly, a paired t-test was used to assess changes in perception of workplace safety before and after PSWT. The results showed no statistically significant difference (t = -1.17, *p* = 0.24). Therefore, the change in perception was not statistically significant. This suggests that perception may require longer-duration or repeated interventions. The results are mentioned in Table 3. When considering the level of perception, 14% of participants were in the higher perception of workplace safety prior to PSWT, and this had increased to 34% after PSWT (see Fig. 1b).

### Regression analysis of gain scores

A generalised linear model (GLM) was employed to analyse the predictive influence of age and gain of perception of workplace safety on the gain of awareness of prevention of workplace violence. The gain scores of both variables were calculated by subtracting the pretest scores from the posttest scores. The model was selected based on the characteristics of the dataset and the presence of negative values in the data. The dependent variable (gain of awareness of prevention of workplace violence) is non-normally distributed and includes negative values; hence, a GLM with normal distribution and identity link function was opted for the analysis. The omnibus test of model coefficients indicated that the model significantly improved prediction over the intercept-only model, *χ²(2)* = 22.011, *p* < 0.001, suggesting that the included predictors explained a significant portion of the variance in the gain of awareness. Wald chi-square tests showed that age was a statistically significant predictor of gain in awareness (Wald *χ²* = 19.627, *df* = 1, *p* < 0.001). The positive regression coefficient (*B* = 0.775, 95% CI [0.432, 1.118]) indicates that for each additional year of age, the gain in awareness of prevention of workplace violence increased by 0.775 units, controlling for gain in perception of workplace safety. Meanwhile, gain in perception of workplace safety is not a significant predictor (Wald *χ²* = 0.038, df = 1, *p* = 0.846), with a non-significant regression coefficient (*B* = -0.002, 95% CI [-0.017, 0.014]), indicating that changes in perception of workplace safety had no significant impact on changes in awareness of prevention of workplace violence.

**Figure:**
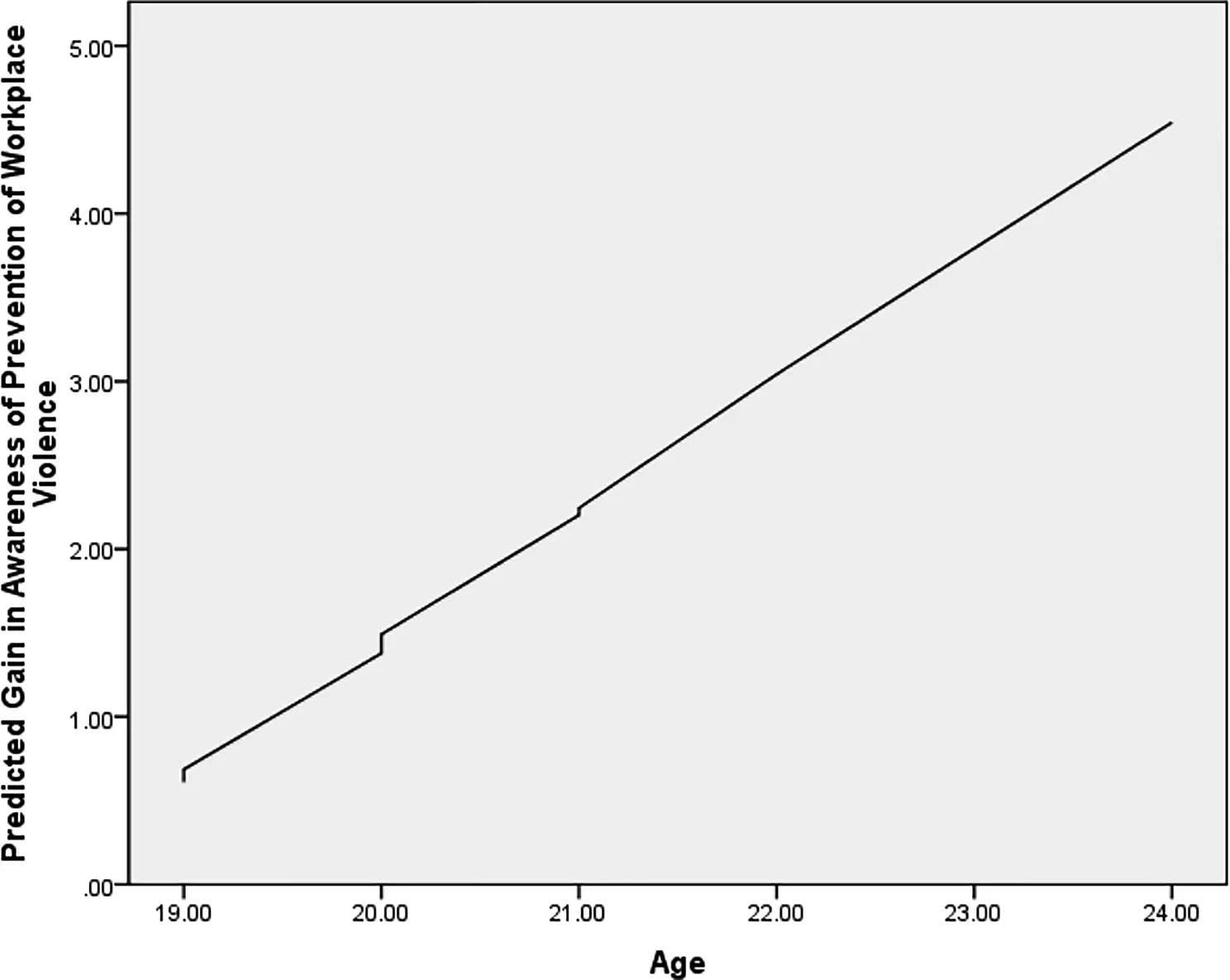

## Discussion

The present study examined the effectiveness of Pro-Safe Work Training (PSWT) in improving awareness and perception of workplace violence among nursing students. The findings demonstrated that PSWT significantly improved awareness of workplace violence prevention among nursing students, while changes in perception of workplace safety were not statistically significant, despite a positive trend. The high baseline awareness observed among the participants suggests that they had a prior theoretical exposure through their nursing curriculum; however, the observed improvement following the PSWT highlights the importance of structured, experiential training in reinforcing and translating knowledge into practice. These findings also underscore the gap between theoretical knowledge and practical preparedness in the prevention of workplace violence.

Previous studies have reported similar gaps in preparedness among nursing students. For instance, a study conducted among nursing students enrolled in psychiatric courses found that the majority had not received structured training in workplace violence prevention, and only a small portion felt confident in handling aggressive and violent situations [33].

The majority of participants demonstrated good knowledge during the pretest, indicating that they had already been exposed to the topic during the curriculum implementation of their BSc Nursing syllabus. The absence of participants in the lower awareness category indicates a strong baseline understanding prior to the intervention. This may reflect the effectiveness of existing academic training; however, such levels of baseline awareness may not be consistent across all nursing education settings. A study conducted in Bangladesh reported that 68% of nurses had good knowledge of workplace violence prevention and management [34]. Similarly, Jeong & Lee, (2020) reported significant knowledge among the nursing students regarding workplace violence prevention [35].

The significant improvement in awareness observed in the present study may be attributed to the experiential and skills-based design of PSWT. The integration of role-play, simulation, and behavioural rehearsal promotes active engagement and enhances knowledge retention, consistent with experiential learning theory. Evidence suggests that interactive training approaches are more effective than passive instruction in improving workplace violence management competencies among nursing students and healthcare workers [35]. These findings reinforce the importance of integrating experiential training methods into nursing education to enhance both theoretical and applied knowledge.

The findings related to perception differed from awareness outcomes, as no statistically significant improvement was observed, although a positive trend was noted. This may be due to the complex and multidimensional nature of safety perception. Unlike awareness, perception is shaped by organizational culture, prior experiences, perceived support, and psychological factors. Literature regarding safety climate suggests that individual perception is more strongly influenced by systemic and environmental factors than by short-term educational interventions [11, 30]. Several factors may explain the lack of statistically significant change. First, the short duration of the intervention may be insufficient to influence deeply held perceptions. Second, the absence of follow-up limited reinforcement of learning. Third, the perception is shaped contextual and psychological factors such as institutional safety climate, hierarchal dynamics and prior experiences which are not easily modified through short duration interventions. A cross-sectional study was conducted in Italy in 2015–2016 among 9607 nursing students across 27 universities reported that the majority of students had a moderate to high perception of workplace safety, supporting the baseline findings of the present study [36].

A study conducted among 130 nurses at a tertiary hospital in Suzhou, demonstrated that structured intervention significantly reduced workplace violence, indicating that practical training can improve behavioural outcomes, although perceptual changes may require sustained exposure [37]. A contrasting study conducted among 147 emergency department staff at a trauma centre in Colorado reported consistently high safety perception irrespective of demographic variables, suggesting that perception may be more strongly influenced by institutional safety climate than individual-level interventions [38]. However, in the present study, the proportion of the participants with high perceptions changed from 14% to 34% indicate a positive directional change suggesting that repeated or sustained interventions may have the potential to improve the perception over time.

This study also aimed to determine the effects of a violence prevention training program (pro-safe work training). There is a need for more interventions, as few structured training programs are currently available, creating a severe practical gap [39]. A study conducted by Cai et al., (2023) [37] to enhance the communication self-efficacy, problem-focused coping style, emotion-focused coping style, and the ability to cope with violence among nursing students showed significantly higher posttest scores after the violence prevention training program.

The regression analysis further supports the findings by demonstrating that age was a significant predictor of improvement in awareness. The findings suggest that older students may benefit more from the training interventions, possibly due to greater cognitive maturity or prior clinical experience. In contrast, changes in perception did not significantly predict awareness gain, reinforcing the distinction between knowledge acquisition and perception, which may require different intervention strategies.

From a practical perspective, the findings of the present study have implications for multiple stakeholders. For nurse educators, simulation, role-play, and scenarios-based training can enhance preparedness to manage workplace violence. For academicians, embedding structured workplace violence prevention modules may strengthen the knowledge and skill acquisition of the undergraduate nursing students. For healthcare administrators, reinforcing safety policies, establishing accessible reporting system may foster safety climate and improve safety perceptions in the institutions. Periodic refresher training may support the sustained behavioural and perceptual change.

### Limitations

The study has several limitations. The one-group pretest-posttest design without a control group limits the ability to attribute observed changes solely to the intervention. The use of purposive sampling technique and single-institution setting restrict generalizability. Additionally, reliance on self-reported measures may introduce response bias. The absence of long-term follow-up further limits the ability to assess retention of knowledge and sustained changes in perception.

## Conclusion

The study demonstrates the effectiveness of the PSWT program among nursing students. Although the PSWT significantly improved awareness regarding the prevention of workplace violence, it did not lead to a statistically significant change in participants’ perception of workplace safety. The results of this study also suggest that while awareness can be increased through educational interventions, changing deeper perceptions may require more comprehensive, continuous practical exposure and systemic administrative support. These findings support the integration of structured workplace violence prevention training into nursing curricula, along with institutional strategies to enhance safety perception.

## Supplementary Information

Below is the link to the electronic supplementary material.

Supplementary Material 1

## Data Availability

Data will be provided upon request.
