# Supplementary material for: Effectiveness of Pro-Safe Work Training (PSWT) and perception of workplace safety among undergraduate nursing students: a mental health perspective
Source: BMC Nurs. 2026 Jun 23;25:802. doi: 10.1186/s12912-026-04930-3 (PMC13543419; doi:10.1186/s12912-026-04930-3)
Supplement: Supplementary file 1 [file 12912_2026_4930_MOESM1_ESM.pdf]

Background proforma

Instructions to the participants: Please read the items and put a tick mark **(√)** against the appropriate response, please respond to all the items.

Personal information:

1. Age in years: ---------
2. Gender:
    a. Male
    b. Female
    c. Transgender
3. Duration of completed clinicals hours:
4. Type of hospital which you are doing your clinicals:
    a. Government medical college hospital
    b. Private medical college hospital
    c. District hospital
    d. private hospitals
    e. nursing homes
5. Residential status
    a. Day scholar
    b. Hostelite
6. Area of residence:
    a. Urban
    b. Rural
7. Type of nursing college you are studying:
    a. Government:
    b. Private:
8. Have you received any previous formal training on workplace safety at hospital?
    a. Yes
    b. No
    if yes, specify
9. Have you received any previous formal training on violence prevention at hospital?
    a. Yes
    b. No
    if yes, specify

10. Are you prepared to handle any workplace violence?
    a. Yes
    b. No
11. have you ever been a victim of workplace- violence?
    a. Yes
    b. No

if yes, specify what was the type of workplace- violence and how did you handle it?

12. Do you work in shifts?
    a. Yes
    b. No

Tool II: Awareness questionnaire for undergraduate nursing students towards the prevention of workplace- violence and improving safety

Kindly go through the following items and place a tick mark in the appropriate place you prefer.

**Sl. No.**	**Statements**	**True**	**False**	**Don't know**
**1.**	Workplace violence is any behaviour that threatens the safety or well-being of employees in the hospital setting			
**2.**	Workplace violence in hospitals encompasses to physical assault, verbal abuse, threats and harassment (e.g.: bullying, stalking, racial, religious, psychological harassment)			
**3.**	Workplace violence in hospitals can involve not only direct acts of aggression but also indirect action that create a hostile work environment			
**4.**	Workplace violence is a common occurrence in healthcare settings			
**5.**	Overcrowding and long waiting time are common risk factors contributing to workplace violence in healthcare settings			
**6.**	Healthcare professionals working in emergency departments are at higher risk of experiencing workplace violence			

**7.**	Compared to healthcare personnel who interact directly with patients, people in administrative roles are not as likely to experience workplace violence.			
**8.**	Patients with a history of substance abuse or mental illness or stress are more likely to engage in violent behaviour in hospitals.			
**9.**	Workplace violence in hospitals can be caused by visitors or by the patients at anytime			
**10.**	Workplace violence in hospitals mainly occurs during regular working hours and not during evening or weekends.			
**11.**	It is important for healthcare professionals to get themselves trained to deal with workplace violence			
**12.**	Every healthcare facility should have safety protocols to prevent workplace violence			
**13.**	Hospital administration should give free of cost support services to the victims of workplace- violence.			
**14.**	De-escalating techniques are ineffective in managing potentially violent persons in healthcare settings			
**15.**	Workplace violence prevention and safety training are only required for novice nurses/ trainee nurses			
**16.**	Nurses are responsible for initiating de- escalating techniques in potentially violent situations			
**17.**	Workplace violence have long term psychological impacts on healthcare professionals			
**18.**	Collaboration with other healthcare professionals is important in addressing workplace violence			

**19.**	Workplace violence can occur in any healthcare setting, including hospitals, clinics and long term care facilities			
**20.**	It is acceptable for healthcare professionals to ignore or downplay incidents of verbal abuse from patients or visitors			
**21.**	Press and media persons can enter the hospital without prior permission			
**22.**	Workplace violence incidents are less common in healthcare settings compared to other industries			
**23.**	Workplace violence can occur between colleagues in addition to patients or visitors			
**24.**	Nurses need to document only the workplace- violence incidents caused by the patients under treatment			
**25.**	Workplace violence incidents do not need to be reported if they do not result in physical harm to anyone			
**26.**	Nurses need not report all the incidents which affects workplace safety to the appropriate authorities			
**27.**	Security personnel are only responsible for reporting incidents of workplace violence			
**28.**	Ignoring incidents of workplace violence can lead to increased risk for future incidents			
**29.**	Nurses should intervene if they witness a colleague experiencing workplace violence			
**30.**	There is an active complaint redressal procedure for workplace violence in the hospital.			

TOOL III: PERCEPTION OF WORKPLACE SAFETY LIKERT RATING SCALE

Instructions: Tick against the items **(√)**.

Sl no	Statements	Strongly disagree	Disagree	Neutral	Agree	Strongly agree
1.	I feel safe from physical harm in my workplace					
2.	I feel emergency exits are clearly marked and easily accessible in my hospital					
3.	I feel safety equipment are readily available and well maintained (e.g.: restraints, telephone for emergency calls, surveillance camera) in my hospital					
4.	I feel workplace facilities are maintained to prevent accidents. (e.g.: crisis management team, incident reporting software) in my hospital					
5.	I feel safety protocols for hazardous materials are clearly communicated and followed in my hospital					
6.	I feel access to sensitive areas is not restricted to authorized personnel only (acute psychiatric wards, emergency ward, critical wards) in my hospital					
7.	I feel security personnel are visible and accessible when needed in my hospital					
8.	I feel security cameras are strategically placed to monitor key areas of the hospital					
9.	I feel confident that the hospitals adequately protected against external threats					

10.	I feel that hospital has effective measures in place to prevent unauthorized access (surveillance camera, alarm system)					
11.	I am aware of the hospital's workplace safety policies and procedures					
12.	I feel that workplace safety policies are consistently enforced					
13.	I feel that there is a clear process for reporting safety concerns or incidents in my hospital					
14.	I feel hospital authority actively seeks and addresses feedback regarding workplace safety					
15.	I feel employees are provided with adequate training on workplace safety in my hospital					
16.	I feel psychologically safe in my workplace environment					
17.	I feel that a therapeutic environment need to be maintained before breaking the bad news					
18.	I feel comfortable expressing concerns or ideas without fear of retaliation					
19.	I feel nursing managers and supervisors show genuine concern for employee's well-being					
20.	I feel there are resources available to support employee's mental health and well- being in my hospital					
21.	I feel workplace safety training is provided regularly and effectively in my hospital					
22.	I feel employees are encouraged to participate in safety- related training and education in my hospital					
23.	I feel training materials and resources are easily accessible to all employees in my hospital					
24.	I feel employees are adequately trained to respond to emergencies in my hospital					

25.	I feel there is a culture of continuous learning and improvement regarding workplace safety among staff in my hospital					
26.	I feel there is effective communication between management and employees regarding safety matters in my hospital					
27.	I feel employees collaborate with each other to identify and address safety concerns in my hospital					
28.	I feel feedback from employees regarding safety improvements is actively sought and considered in my hospital					
29.	I feel there is a culture of mutual support and accountability for maintaining workplace safety in my hospital					
30.	I feel team members are encouraged to hold each other accountable for following safety protocols in my hospital					
